# Emergence and spread of *Mycobacterium ulcerans* at different geographic scales

**DOI:** 10.1128/spectrum.03827-23

**Published:** 2024-03-05

**Authors:** Martial Briand, Alexandra Boccarossa, Adrien Rieux, Marie-Agnès Jacques, Line Ganlanon, Christian Johnson, Matthieu Eveillard, Laurent Marsollier, Estelle Marion

**Affiliations:** 1 Univ Angers, Institut Agro, INRAE, IRHS, SFR QUASAV, Angers, France; 2 Univ Angers, CNRS, ESO, Angers, France; 3 CIRAD, UMR PVBMT, Saint Pierre, La Réunion, France; 4 CDTLUB Pobè, Fondation Raoul Follereau, Pobè, Benin; 5 CIFRED, University of Abomey-Calavi, Abomey-Calavi, Benin; 6 INCIT, Inserm, Univ Angers, CHU Angers, Angers, France; Assistance Publique - Hopitaux de Paris, France

**Keywords:** *Mycobacterium ulcerans*, phylogeography, WGS, evolution

## Abstract

The classical lineage of *Mycobacterium ulcerans* is the most prevalent clonal group associated with Buruli ulcer in humans. Its reservoir is strongly associated with the environment. We analyzed together 1,045 isolates collected from 13 countries on two continents to define the evolutionary history and population dynamics of this lineage. We confirm that this lineage spread over 7,000 years from Australia to Africa with the emergence of outbreaks in distinct waves in the 18th and 19th centuries. In sharp contrast with its global spread over the last century, transmission chains are now mostly local, with little or no dissemination between endemic areas. This study provides new insights into the phylogeography and population dynamics of *M. ulcerans,* highlighting the importance of comparative genomic analyses to improve our understanding of pathogen transmission.

**IMPORTANCE:**

*Mycobacterium ulcerans* is an environmental mycobacterial pathogen that can cause Buruli ulcer, a severe cutaneous infection, mostly spread in Africa and Australia. We conducted a large genomic study of *M. ulcerans*, combining genomic and evolutionary approaches to decipher its evolutionary history and pattern of spread at different geographic scales. At the scale of villages in an endemic area of Benin, the circulating genotypes have been introduced in recent decades and are not randomly distributed along the river. On a global scale, *M. ulcerans* has been spreading for much longer, resulting in distinct and compartmentalized endemic foci across Africa and Australia.

## INTRODUCTION

*Mycobacterium ulcerans* (*Mu*) is an environmental mycobacterial pathogen that can cause Buruli ulcer, a severe cutaneous infection associated with a high rate of permanent disability in immunocompetent subjects if treated late (1). Most Buruli ulcer patients live in West and Central Africa or Australia but this disease is found on all continents (2, 3). Comparative genomics has revealed that *Mu* has a clonal population structure, with three distinct lineages (4, 5) (Fig. S1). The most recently diverged lineage (known as the “classical lineage” or “lineage 3”) is the most prevalent clonal group identified in clinical infections; it is widespread in Australia and Africa (4, 5) (Fig. S1). In Africa, two sub-lineages of lineage 3 are distinguished and named MU_A1 and MU_A2, the latter being sporadic and close to strains from Papua New Guinea (Fig. S1).

The transmission of *Mu* between humans has never been demonstrated but various hypotheses have been put forward concerning the mode of transmission of *Mu* from the environment to humans. Inoculation of the skin with *M. ulcerans* is necessary (6). The inferred ancestor of *Mu* is an aquatic mycobacterium, and it is therefore thought likely that *Mu* has an aquatic reservoir (7). *Mu* has been detected by specific qPCR assays in various compartments of the aquatic food chain in swampy areas of Africa (8–14) but very few isolates have ever been obtained from this environment because of the very low growth rates of *Mu* (doubling time: 3.5 days) and high rates of contamination (optimal temperature of growth of 30°C) (14). Links between the aquatic environment and Buruli ulcer cases have been highlighted in various epidemiologic studies performed in Africa over the last few decades (15–17). One route of transmission from the aquatic environment to humans involves aquatic bugs harboring the bacillus and the inoculation through bites (10, 11, 18). Mammals may also serve as a reservoir, although this has to date only been demonstrated in Australia, where a possum species endemic to this continent can act as an asymptomatic carrier with gut colonization or may present cutaneous infections caused by *Mu* (19, 20). There is currently no evidence that any other mammals play a similar role elsewhere in the world (21, 22). In Australia, Buruli ulcer is considered to be a zoonosis, with mosquitoes as the most likely mechanical vector (20, 23).

An understanding of the global evolution of *Mu*’s recent lineage, and its emergence and dissemination at different spatial levels is crucial to better understand *Mu* population dynamics and develop effective control strategies, which remain sorely lacking. However, the complexity and low success rates for *Mu* isolation from clinical samples collected from patients living in the remote rural areas of Africa in which this disease is endemic, together with the absence of *Mu* isolates collected from the environment has complicated traditional epidemiologic investigations. Here, to circumvent these difficulties, we conducted a large genomic study of *Mu* combining genomic and evolutionary approaches to decipher its evolutionary history and pattern of spread at different geographic scales.

## MATERIALS AND METHODS

### Data set 1 Benin

#### Bacterial isolates and DNA sequencing (data set 1)

We performed whole-genome sequencing (WGS) on 119 *M*. *ulcerans* strains isolated from patients diagnosed with and treated for BU during the 2015–2021 period at the *Centre de Diagnostic et Traitement de la Lèpre et de l’Ulcère de Buruli* (CDTLUB) in Pobè, Benin (24). We cultured the isolates on the Lowenstein-Jensen medium for 5 months and then extracted DNA from them as previously described (24). Genome sequencing was performed with the DNBseq short-insert library (PE150) at the BGI platform. The reads generated were submitted to the National Center for Biotechnology Information Sequence Read Archive (Bioproject PRJNA881288). To constitute data set 1, we added reads from a previous study (Bioprojet PRJNA499075) also generated from isolates sampled between 2007 and 2016 in Benin (24).

#### Genome assembly (data set 1)

Reads were assembled with SPAdes (25) (version 3.15.5), using the default k-mer parameters (-k 21, 33, 55, 77, 99, 127) and the following options: --cov-cutoff auto, --isolate (26). Only contigs of 250 bp or longer were retained. We assessed the quality of the genome sequence assemblies with checkm (27) (v1.1.6): we retained only genomes affiliated to checkm marker lineage *Mycobacterium* (UID1816). We exclude also genomes for which more than 15 genes of the *Mycobacterium* marker set were absent, and for which more than 10 genes of this set were present in multiple copies.

#### Core-genome alignment (data set 1)

A core-genome alignment was generated with Parsnp (28) (v1.7.4), using the default parameters. The Agy99 (SAMN02603346) genome was used as the reference strain genome for the *Mu* strains of data set 1. This strain was also used to root the tree.

#### Phylogeographic analysis (data set 1)

We performed a Kulldorf spatial scan in SaTScan 9.6 (29) to investigate the presence and location of spatial clusters identified based on geographic coordinates. We used QGIS 2.10 to generate figures illustrating the geographic distribution of *M. ulcerans*.

#### Identification of SNPs and molecular dating (data set 1)

The recombinant regions of the core genome were identified with clonalframeML (30, 31) and masked in the core-genome alignment with the bedtools maskfasta command. SNP sites were extracted from the masked core-gene alignment with snp-sites (32) and a non-recombining phylogeny was built with RaxML 8.2.4 (33), using a rapid bootstrap analysis and a general time-reversible model of evolution according to a distribution with four rate categories (GTRGAMMA). The maximum-likelihood (ML) tree obtained for strains with a known date of isolation (*n* = 315), spanning 15 years of evolution (2006–2021), was used to investigate the possible presence of a temporal signal, a prerequisite for tip dating inferences (34, 35). For this purpose, we ran Phylostems (36) to compute the linear regression between sample age and root-to-tip distances at every internal node of the tree. Nodes displaying both a significant linear regression and a positive slope were assumed to contain detectable amounts of evolutionary change and were considered to correspond to a suitable evolutionary scale for tip dating. We performed tip-dating inferences with BEAST v1.8.4 (34, 37). The nucleotide substitution rate was modeled with a general time-reversible (GTR) substitution model of evolution. Between-site rate variation was modeled with a discrete gamma distribution with four rate categories. We assumed an uncorrelated lognormal relaxed clock to account for rate variation between lineages. The contribution of prior assumptions about demographic history was minimized by the use of an extended Bayesian skyline plot approach to integrate data over different coalescent histories. The tree was calibrated with tip dates only. We ran five independent chains in which samples were drawn every 5,000 Markov chain Monte Carlo steps from a total of 50,000,000 steps, after a discarded burn-in of 5,000,000 steps. Convergence to the stationary distribution and sufficient sampling and mixing were checked by inspecting posterior samples (effective sample size >200). Parameters were estimated by combining samples from the different chains. The best-supported tree was estimated from the combined samples with the maximum clade credibility method implemented in TreeAnnotator (34). Rate-dating calibration (with a relaxed log-normally distributed molecular clock) was performed at evolutionary scales at which no temporal signal was detected with BEAST v1.8.4. Rather than specifying tip dates, we used a prior for the substitution rate by drawing values from a normal distribution with mean and standard deviation values fixed at those inferred during the tip-dating calibration.

### Data set 2 Australia and Africa

#### Origin of *M. ulcerans* reads (data set 2)

We computed a second global/worldwide data set including the genomes from data set 1 and deposited *Mu* reads for Bioprojets PRJEB4025, PRJNA163311, PRJNA313185, and PRJNA421048. We also worked with six previously assembled genomes (ATCC19423: GCA_022374915.1; CSURQ0185: GCA_902506705.1; JKD8049: GCA_020616615.1; Agy99: GCA_000013925.1; S4018: GCA_001870585.1; and SGL03: GCA_900638745.1). All assembled genomes of Data set 2 are available on Bioproject PRJNA1008572. Information about all the isolates used is provided in Table S1.

#### Genome assembly (data set 2)

We carried out an adapter-cleaning step on *Mu* reads with trimGalore (26) (version 0.6.6) before running the same assembly process as for data set 1.

#### Identification of specific sequences (data set 2)

We used Skif2 software (https://sourcesup.renater.fr/wiki/skif2/) (30) with a k-mer length of 22 to identify the sequences specific to each sub-lineage or clade. The genome of *M. marinum* is not included in this analysis. Each sequence of the ingroup was split into k-mers of length 22. We then searched the outgroup sequences for the k-mers present in all the sequences of the ingroup; only k-mers absent from all outgroup sequences were retained. These specific k-mers were mapped onto the sequence of one of the ingroup genomes and concatenated if they were consecutive.

#### Core-genome alignment (data set 2)

A core-genome alignment was generated with Parsnp (28) (v1.7.4), using the default parameters. The *M. marinum* 1218R (SAMN07811443) genome was used as the reference strain genome for the *Mu* strains of data set 2. This strain was also used to root the tree.

#### Identification of SNPs and molecular dating (data set 2)

SNP identification was performed as described above for data set 1. Molecular dating of data set 2 (*n* = 1,045) was performed using a rate-dating strategy (using the same parameters as specified for data set 1) because sampling dates were not available for all sequences, hence impeding a thorough investigation of the temporal signal and the use of a tip-dating approach. Hence, all sequences were considered isochronous and contemporary. Because we failed to reach convergence on the whole data set (probably due to a too large data set), we used Treemmer (38) to reduce our data set with minimal loss of diversity. A new rate-dating inference was then performed with BEAST v1.8.4 on a subsampled data set of 300 *Mu* isolates (Table S1) using a normal distribution with a mean equal to 7 × 10^−8^ as prior to the substitution rate and a log-normally distributed relaxed molecular clock.

## RESULTS

### Evidence of a strong spatial structure at a local scale (data set 1)

**Fig 1:**
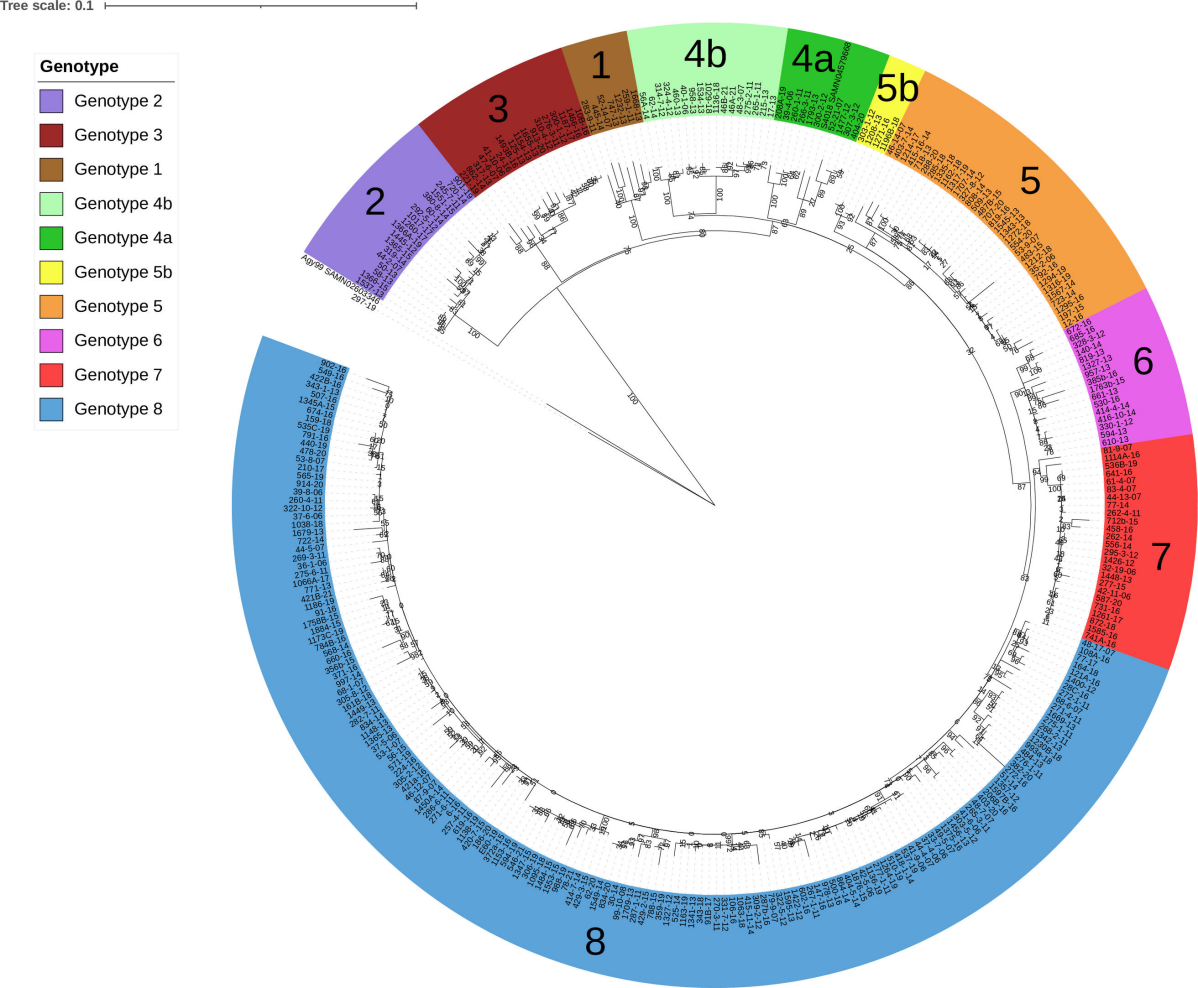
Core-genome phylogeny of 307 *M*. *ulcerans* strains (Mu-A1 lineage) isolated from patients living in South-East Benin and South-West Nigeria. raxML phylogenetic tree of *M. ulcerans* isolates inferred from 1,520 core-genome SNP analysis by Parsnp and visualized with iTOL. Analyses were run using RAxML with a rapid 1,000 bootstrap analysis and a general time-reversible model of evolution according to a distribution with four rate categories (GTRGAMMA). The reference genome (Agy99) is shown. Based on the segregation indicated by this tree, the genomes were split into 10 monophyletic or paraphyletic genotypes. A specific color is assigned to each taxon.

We previously conducted a first phylogenetic analysis at a local scale, using *Mu* genomes sampled from South-East Benin and South-West Nigeria (24). We identified eight different genotypes within the Mu-A1 lineage and showed that some of these genotypes had a strong local spatial structure. Since 2018, we have obtained the sequences of an additional 119 new *Mu* strains isolated from patients of the same geographic origin, resulting in a set of 326 genomes of human origin (2006–2021) from this geographic area. We constructed a new phylogenetic tree after the *de novo* assembly and alignment of all the genomes. From this initial analysis, we eliminated five strains due to insufficient assembly quality and 13 strains due to their large distance, reducing the size of the core genome. We identified 1,520 SNPs and obtained a similar general genetic structure, including the eight previously defined genotypes (Bootstrap values > 70%) (Fig. 1). The strains from the first study were generally assigned to the same genotype in this analysis, but two new groups (4b and 5b) were defined in this analysis. The “297–19” strain was found to be much closer to the Agy99 strain than to the other strains analyzed here. This observation led to the patient carrying this isolate being interviewed. He declared that he had traveled no farther than 20 km from home since his birth.

**Fig 2:**
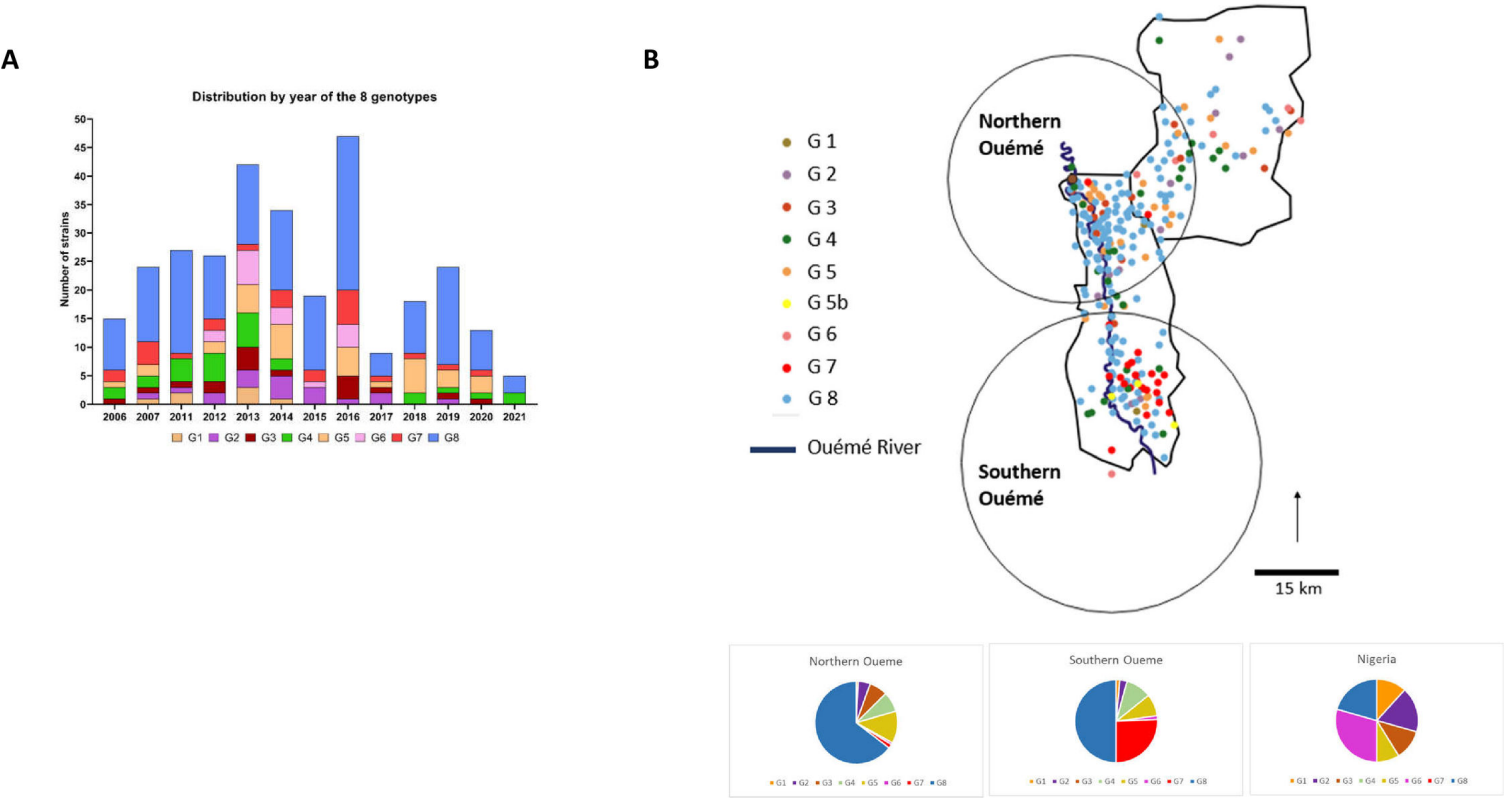
Temporal and spatial distribution of the eight genotypes. (A) Distribution of *Mycobacterium ulcerans* genotypes according to diagnosis dates for Buruli ulcer patients in Benin and Nigeria. The distribution of genotypes was tested with contingency tables (Fisher’s exact test) comparing years and there is no significant difference. (B) Spatial cluster detection results for *Mycobacterium ulcerans* genotypes for Buruli ulcer patients in Benin. Two significant areas were detected along the Ouémé River (northern Ouémé and southern Ouémé). Composition of these two clusters relative to that expected for a random distribution.

**Fig 3:**
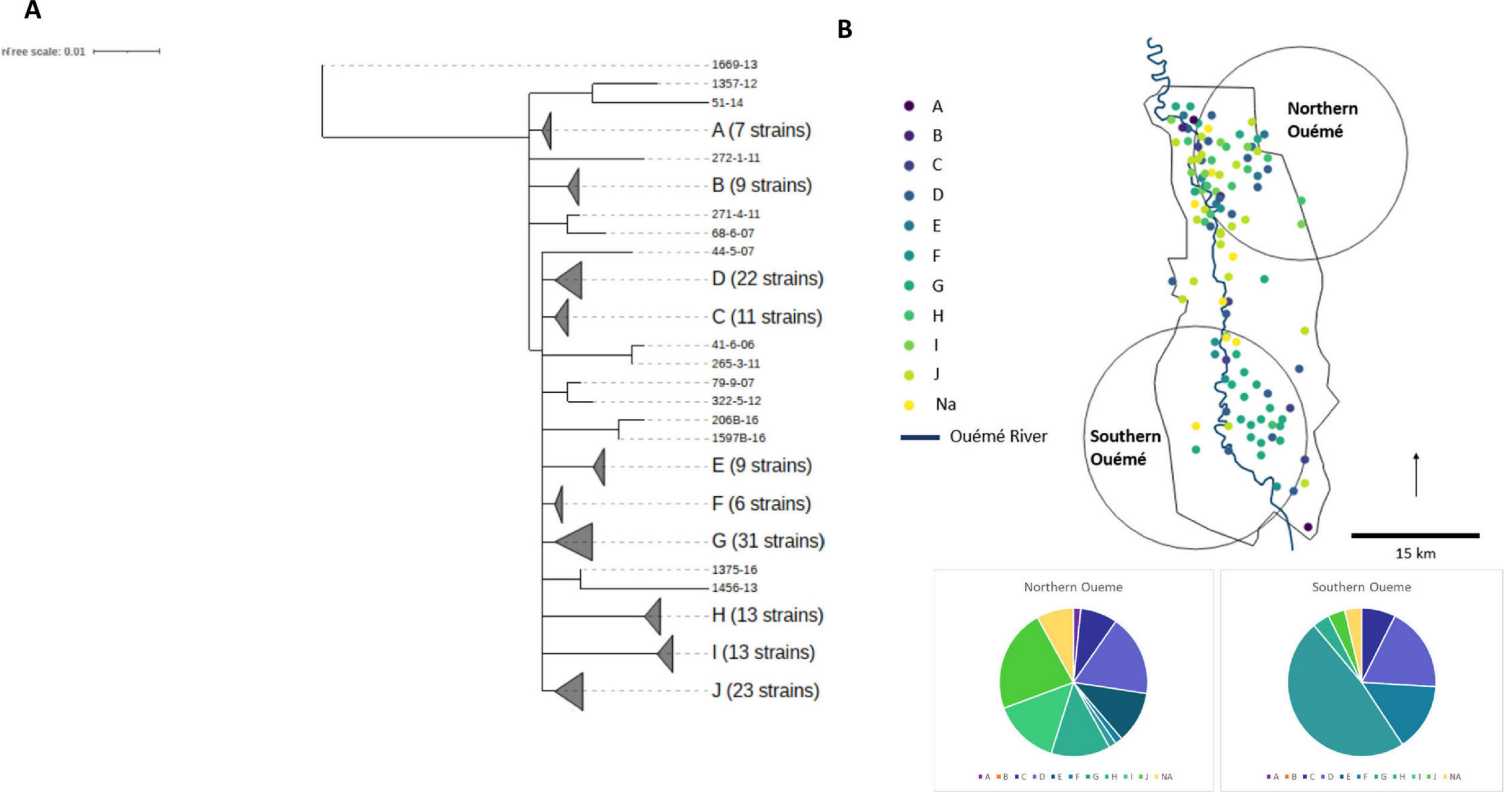
Focus on genotype 8, its subgenotypes, and spatial distribution. (A) The phylogenetic tree for genotype 8 reveals the presence of potentially emerging genotypes. (**B**) Location of the clusters of subgenotypes. (B) Two significant spatial clusters were identified in which specific subgenotypes were overrepresented relative to other areas. Composition of these two clusters relative to that expected for a random distribution.

We found no association between genotype and year of strain isolation over a period of 15 years (Fig. 2A). About half the isolates belonged to genotype 8 and the rest belonged to one of the other genotypes (1–7) in various proportions over the time period considered (Fig. 1 and 2A). Focusing on genotype 8, 159 strains belonged to this genotype, and we were able to distinguish 10 subgenotypes (8.A to 8.J) corresponding to 118 of these strains (Fig. 3A). Four additional subgenotypes have been identified since the previous study, and 16 strains not previously assigned to a particular subgenotype were assigned to one of the four new subgenotypes.

We observed a non-random distribution of some genotypes in the geographic area studied. Indeed, genotype 7 and subgenotype 8.G appeared strongly localized to the south of the Oueme River, whereas the other subgenotypes of genotype 8 were mostly concentrated in northern Oueme, with subgenotypes 8.E, 8.H, 8.I, and 8.J specific to this area (Fig. 2B and 3B; Tables 1 and 2). Genotypes 1, 2, 3, and 6 remained highly specific to Nigeria (Table 1). These results confirmed previous observations based on fewer genotypes in the same geographic areas (24).

**TABLE 1:** Spatial clustering of genotypes 1 to 8 and relative risks

Cluster	Nb. of strains	Radius	*P* value^*a*^	G1	G2	G3	G4	G5	G5b	G6	G7	G8
Northern Oueme	147	20.30 km	0.00027	0.17	0.55	1.45	0.94	1.75	0	0.14	0.092	1.56
Southern Oueme	82	24.6 km	1.2 × 10^−7^	0.43	0.35	0	0.91	0.29	Infinity	0.37	28.71	0.97
Nigeria	40	76 km	4.7 × 10^−8^	8.53	5.69	3.49	0	0.71	0	14.08	0	0.35

^*a*^ Kulldorf spatial scan statistics.

**TABLE 2:** Spatial clustering of sub-genotypes of genotype eight and relative risks

Cluster	Nb. of strains	Radius	*P* value	G8A	G8B	G8C	G8D	G8E	G8F	G8G	G8H	G8I	G8J	G8NA
Northern Oueme	72	11.27 km	1.10 × 10^−4^	0.19	0.14	1.15	1.15	infinity	0.23	0.13	2.59	5.19	2.16	1.32
Southern Oueme	32	11.78 km	9.3 × 10^−4^	0	0	0.96	1.13	0	7.69	5.03	0.32	0	0.17	0.59

### A recent temporal signal in the genotypes specific to Benin (data set 1)

**Fig 4:**
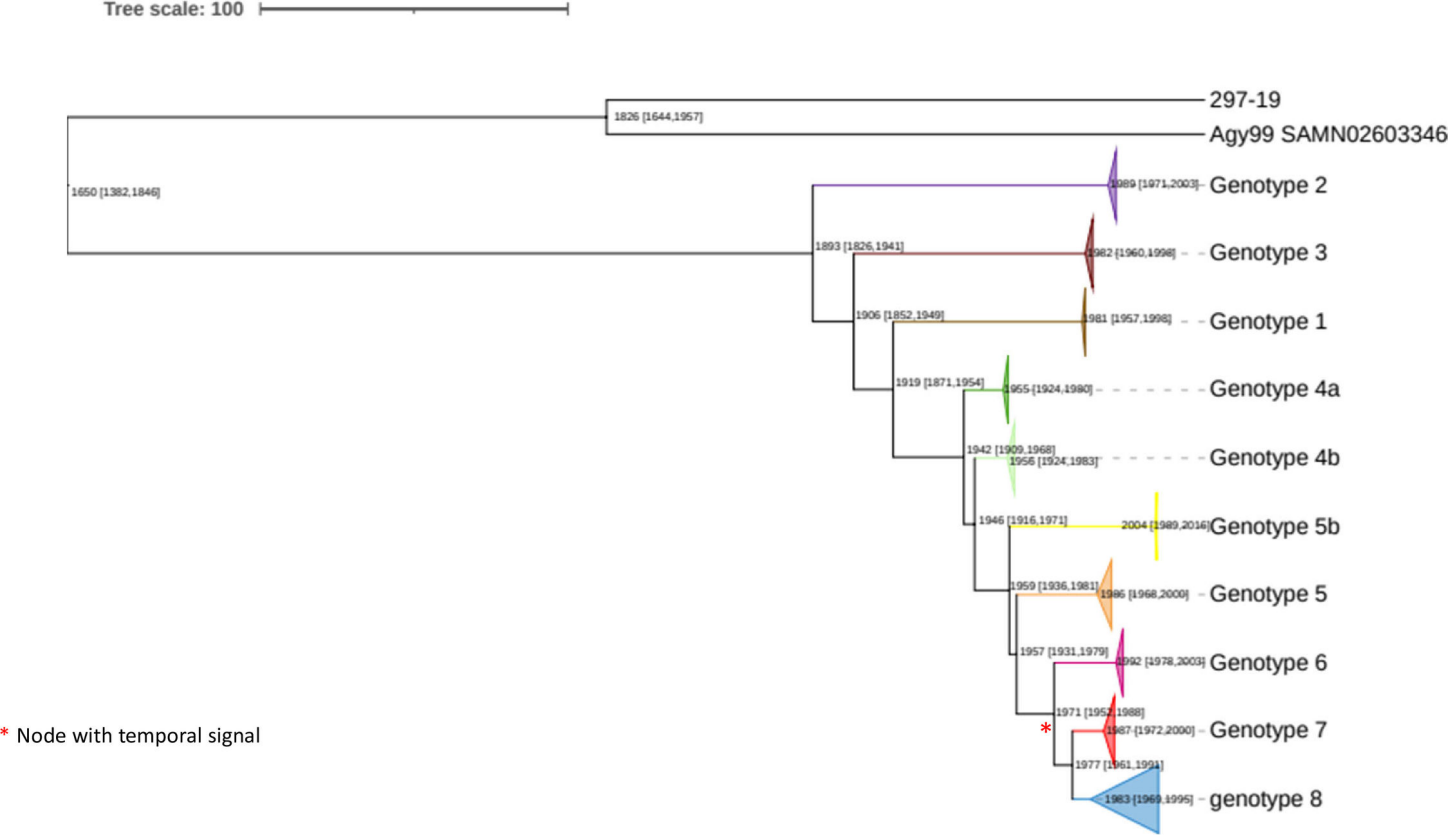
Time tree of the eight genotypes (**G1–G8**) of data set 1. BEAST-dated phylogenetic tree of strains built from non-recombining SNPs. Mean node ages and their associated 95% highest posterior density intervals are indicated in calendar years. The branches of the different genotypes are collapsed and colored. The red star indicates the clade at which a significant temporal signal was found and for which tip dating was performed.

We used Phylostems with the core SNP ML phylogeny to investigate the evolution of the 307 *Mu* strains over time. A sufficiently strong temporal signal (i.e., a detectable amount of evolutionary change) for the analysis was detected at the node corresponding to the most recent common ancestor (MRCA) of genotypes 6, 7, and 8 (*P*-value = 0.0149) (Fig. 4). Multiple factors may account for the absence of a significant temporal signal in the other clades. These factors include overall genetic structure, a limited set of samples, and the short time interval between the dates of collection and the MRCA in each clade. Tip-dating inference with BEAST at this evolutionary scale made it possible to infer (i) a mean substitution rate of 7.258 × 10^−8^ mutations per site per year (95% HPD: 4.5979 × 10^−8^–9.8714 × 10^−8^), corresponding to about 0.33 mutations per genome and per year and (ii) a date of 1965 for the MRCA of genotypes 6, 7, and 8 (95% HPD: 1766–1993). We dated the divergence times of the other genotypes within the whole tree, by performing rate dating, with integration of the mean rate of substitution from the tip-dating calibration. This method yielded an estimated date for the MRCA of all eight genotypes of 1893 (95% HPD: 1826–1941) and the age of all the other nodes in the tree (Fig. 4). Genotypes 7 and 8, which were specific to the Oueme River area of Benin, seemed to have evolved recently (1977, 95% HPD 1961–1991), with an estimated date of 1987 for the emergence of genotype 7 (95% HPD: 1972–2000) and 1983 for genotype 8 (95% HPD: 1969–1995).

We then performed comparative genomics, contrasting the genotype 1–8 isolates with other *Mu* strains belonging to the “classical lineage 3 of *M. ulcerans*” isolated from various countries worldwide (4, 5), with the aim of (i) identifying the position of the 13 isolates removed from data set 1 due to their large genomic distances; (ii) observing the position of the data set 1 isolates with respect to other isolates on a phylogenetic tree; (iii) detecting the presence of specific acquisitions/deletions in the G1–G8 isolates.

### The classical lineage of *Mu* comprises three sub-lineages worldwide and five distinct clades in Africa (data set 2)

**Fig 5:**
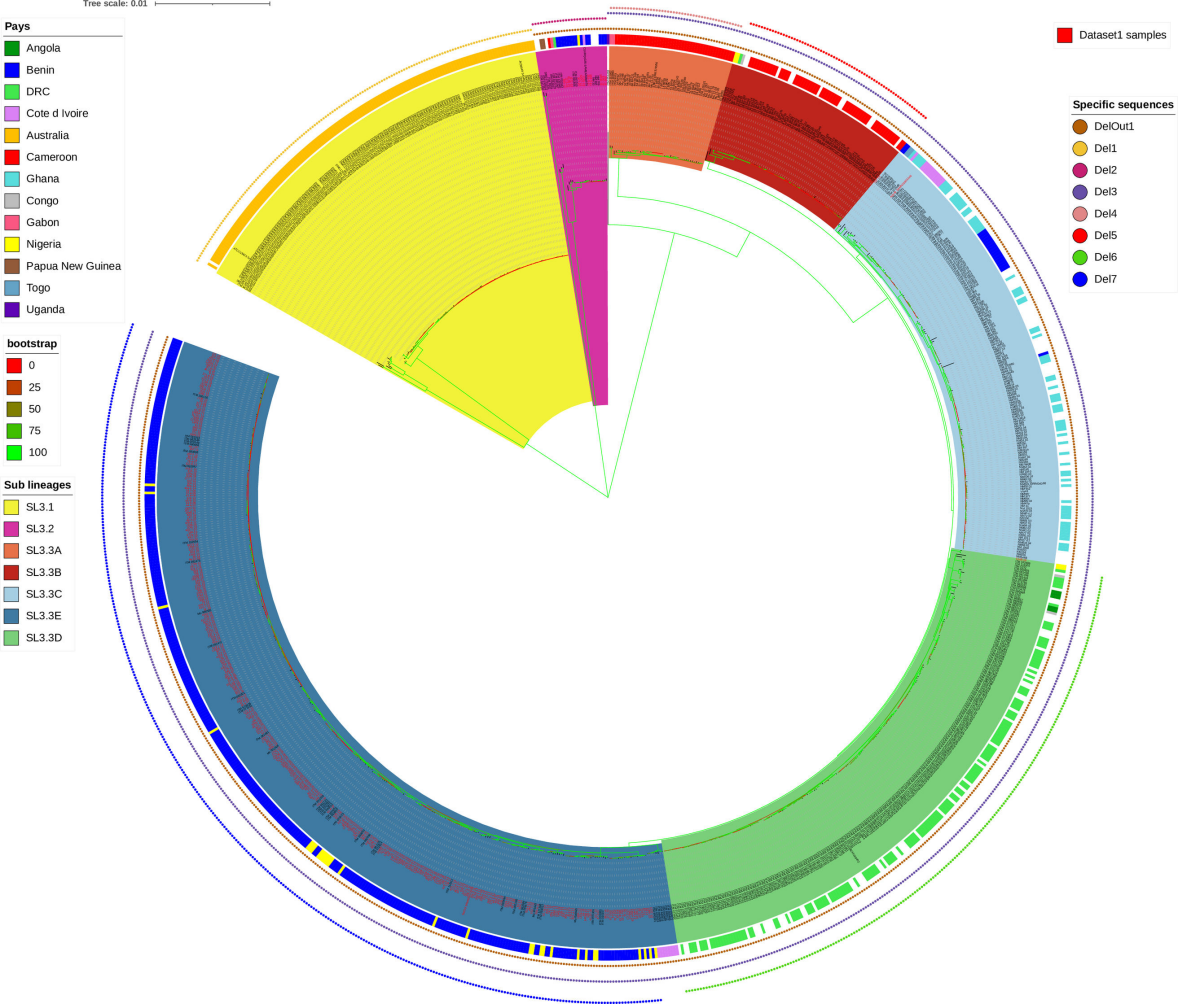
The recent Mu lineage is composed of three sub-lineages, including five clades in the African sub-lineage. Core-genome phylogeny of 1,045 *M*. *ulcerans* strains isolated from patients living in Australia, Africa, and Papouasie New Guinea based on 16,500 SNPs. Analyses were run using RAxML with a rapid 1,000 bootstrap analysis and a general time-reversible model of evolution according to a distribution with four rate categories (GTRGAMMA). The *M. marinum* outgroup was not represented. The presence of a specific deletion for each genome is indicated by the colored dot corresponding to this specific sequence and is also reported in Table 4. The geographic origin of the patients is indicated by a line of color. A clade color identifies the SL3.1 and SL3.2 sub-lineages and five clades in the SL3.3 sub-lineage. Strains whose names are written in red are data set 1 strain.

**Fig 6:**
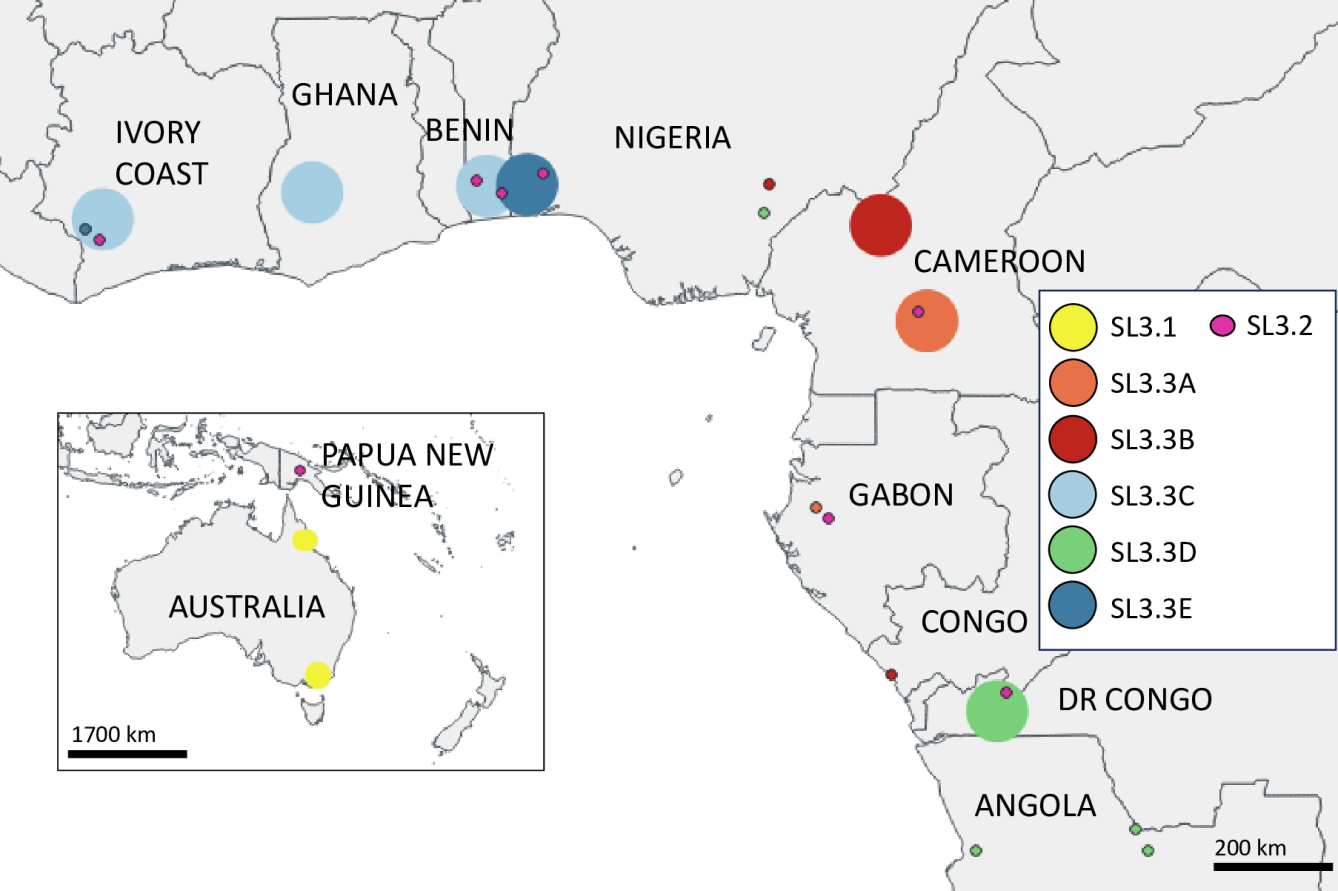
Localization of the sub-lineages and clades of *Mu’*s recent lineage in West Africa and Australia. The size of the circles is not proportional to the sample size.

We analyzed 1,045 genomes obtained from clinical isolates from 13 countries on two continents (11 African countries, Australia, and Papua New Guinea). These isolates covered a time span of 76 years (from 1945 to 2021) of evolution. *Mu* genome size ranged from 5.18 to 5.81 million base pairs. We investigated the phylogenetic diversity of the classical lineage of *Mu* by building a maximum-likelihood phylogeny and identified three sub-lineages, named SL3.1, SL3.2, and SL3.3 (Bootstrap >90%) (Fig. 5A). Within the sub-lineage SL3.3, we can distinguish five clades, named SL3.3A to SL3.3E (Bootstrap value >75%) (Fig. 5A). The mean genetic distance between the three sub-lineages was 3,741 ± 2,804 whole-genome SNPs (Table 3). The genetic distance within each group ranged from 164 SNPs for SL3.3B to 4,299 SNPs for SL3.2. All the sub-lineages and clades were well represented and closely linked to the geographic origin of the isolates, except SL3.2 (Fig. 6). SL3.1 was present in Australia, while SL3.3 was present in Africa. Inside SL3.3, SL3.3A is mainly represented by strains isolated from patients of the Cameroon-Nyong area, SL3.3B of the Cameroon-Mape area, SL3.3C of Ghana, Côte d’Ivoire and the western part of Benin, SL3.3D in the Democratic Republic of Congo, and SL3.3E in the eastern part of Benin and the western part of Nigeria (Table 4). Some atypical geographically inconsistent strains were found at the first node of each of the five clades. Finally, no spatial structure was observed for SL3.2, which contained isolates from Papua New Guinea and different countries in Africa, the last ones corresponding to the Mu-A2 sub-lineage, whereas the 5 clades of SL3.3 could be grouped as the Mu-A1 sub-lineage. Twelve of the 13 strains not included in data set 1 for Benin belonged to SL3.2. The remaining strain excluded from the initial analysis (124B-16) was an atypical isolate at the first node of the SL3.3D clade.

**TABLE 3:** SNPs between strains of the sub-lineages and clades^*a*^

	SL3.1	SL3.2	SL3.3A	SL3.3B	SL3.3C	SL3.3D	SL3.3E	*M. marinum*
SL3.1	942	7317	5047	4975	5738	5976	6120	40915
SL3.2		4299	7583	7507	8269	8507	8640	43700
SL3.3A			374	1205	1969	2207	2355	41425
SL3.3B				164	1321	1560	1707	41349
SL3.3C					989	2296	2443	42093
SL3.3D						1223	2670	42341
SL3.3E							1344	42482
*M. marinum*								0

^*a*^ Dark gray boxes represent diversity within a clade.

**TABLE 4:** Specific sequences and specific genomic deletion for each profile compared to the others

SL/clade	Number isolates	Country origin	Name of deletion or specific sequence	Reference strain	Accession ID	Start position	End position	Sequence size	Description of affected genes
SL3.1	152	Australia (150), NA (2)	Del1	Agy99_SAMN02603346	CP000325.1	2685596	2686940	1345	MUL_2400; MUL_2403: pseudogenes
SL3.2-SL3.3		PNG (2) and Africa	DelOut1	ATCC19423_SAMN20254269	CP092429.1	2550325	2550697	373	PPE family protein, pseudogene (ref: ATCC19423)
SL3.2	27	Benin (14), PNG (2), Cameroon (1), Cote d'Ivoire (1), DRC (1), Gabon (1), Nigeria (1), NA (6)	Del2a	Agy99_SAMN02603346	CP000325.1	3194035	3195151	1116	MUL_2868: pseudogene
			Del2b	Agy99_SAMN02603346	CP000325.1	2627821	2628378	558	Insertion sequence: IS2606; MUL_2348: polyketide synthase, IS insertion and frame-shift mutation
			Del2c	Agy99_SAMN02603346	CP000325.1	2871167	2871907	741	MUL_2574: conserved hypothetical membrane protein; MUL_2575: pseudogene
			Del2d	Agy99_SAMN02603346	CP000325.1	2977430	2979451	2022	MUL_2663: pseudogene
SL3.3	848	Africa	Del3a	Agy99_SAMN02603346	CP000325.1	156392	156700	309	MUL_0150: adenylate/guanylate cyclase domain-containing protein
			Del3b	Agy99_SAMN02603346	CP000325.1	241434	242174	741	MUL_0233: cation-transporting ATPase
			Del3c	Agy99_SAMN02603346	CP000325.1	1155654	1156066	413	MUL_1089: hypothetical protein
			Del3d	Agy99_SAMN02603346	CP000325.1	2478515	2479050	536	MUL_2219: IS2606 transposase
SL3.3A	47	Cameroon-Nyong (45), Gabon (2)	Del4a	Agy99_SAMN02603346	CP000325.1	3789939	3809628	19690pb	MUL_3421: cstA; MUL_3422: chaperone protein DnaK1; MUL_3423: short-chain dehydrogenase; MUL_3424: hypothetical protein; MUL_3425: multidrug-transport integral membrane protein Mmr; MUL_3426: transcriptional regulatory protein; MUL_3427: hypothetical protein; MUL_3428: hypothetical protein; MUL_3429: hydrolase; MUL_3430: succinate-semialdehyde dehydrogenase [NADP+] dependent, PutA_2; MUL_3431: fatty-acid-CoA ligase; MUL_3433: hypothetical protein; MUL_3434: hypothetical protein; MUL_3435: oxidoreductase GMC-type
			Del4b	Agy99_SAMN02603346	CP000325.1	3811317	3811851	535 pb	No
SL3.3B	70	Cameroun-Mape (57), Congo (1), DRC (1), NA (11)	Del5	Agy99_SAMN02603346	CP000325.1	5078852	5079378	527 pb	MUL_4580: Carbon monoxide dehydrogenase large chain (CoxL_1)
SL3.3C	169	Ghana (71), Benin-Kouffo (18), Cote d'Ivoire (11), NA (69)	No deletion						
SL3.3D	215	RDC (107), Angola (4), Congo (2), NA (102)	Del6	Agy99_SAMN02603346	CP000325.1	1713076	1713435	360 pb	MUL_1583: hypothetical protein
SL3.3E	347	Benin (318), Nigeria (29)	Del7	Agy99_SAMN02603346	CP000325.1	116556	117036	481 pb	MUL_0115 and MUL_0116: Carbon monoxide dehydrogenase large chain (Cox L) and small chain (Cox S)

### The three sub-lineages have specific sequences (data set 2)

**Fig 7:**
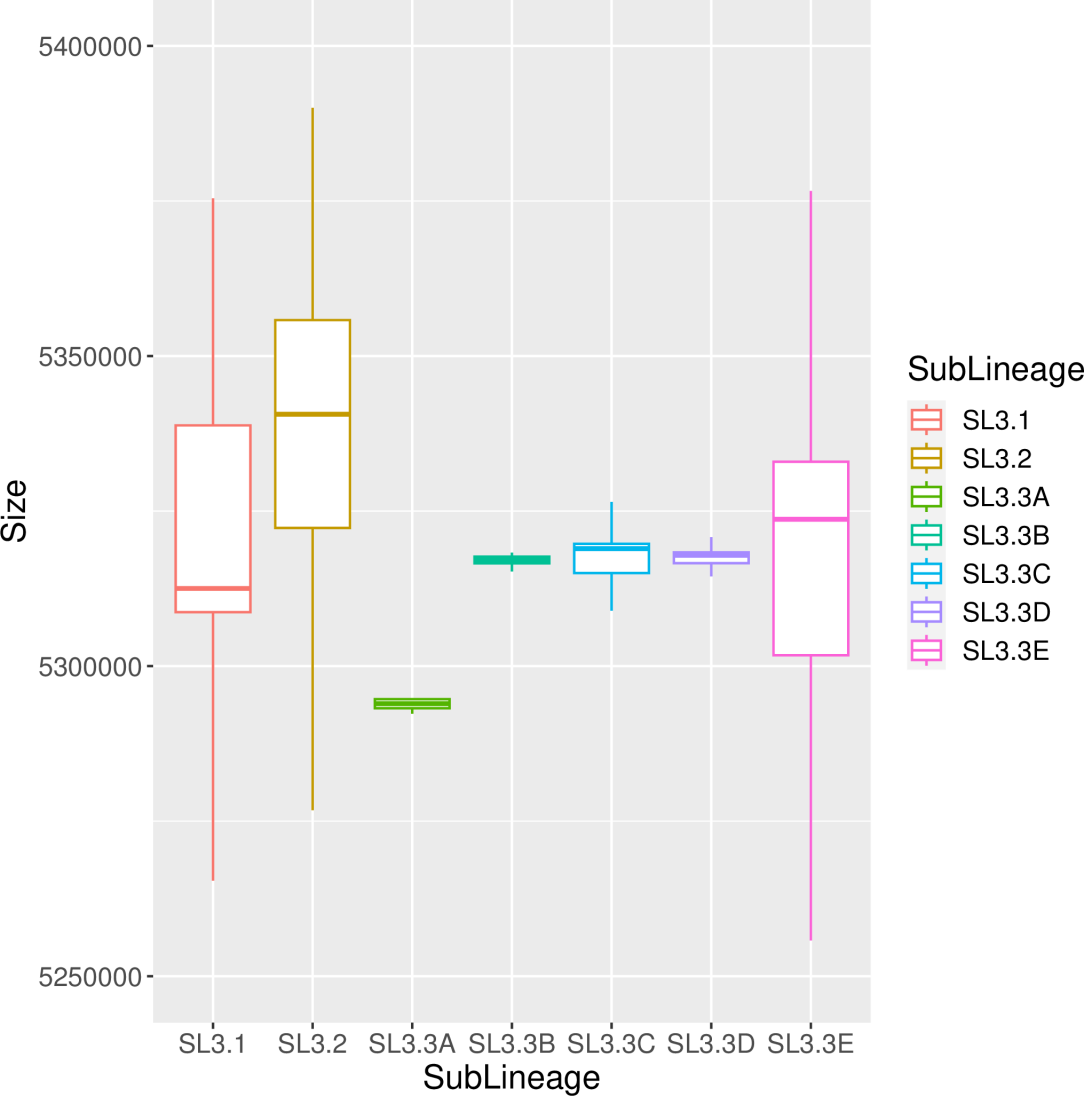
Distribution of the genome sizes according to sub-lineages and clades. The distribution of the size of each aligned genome is observed using a box-and-whisker plot according to their group (Sub-lineages SL3.1, SL3.2, and SL3.3 and clades of SL3.3).

**Fig 8:**
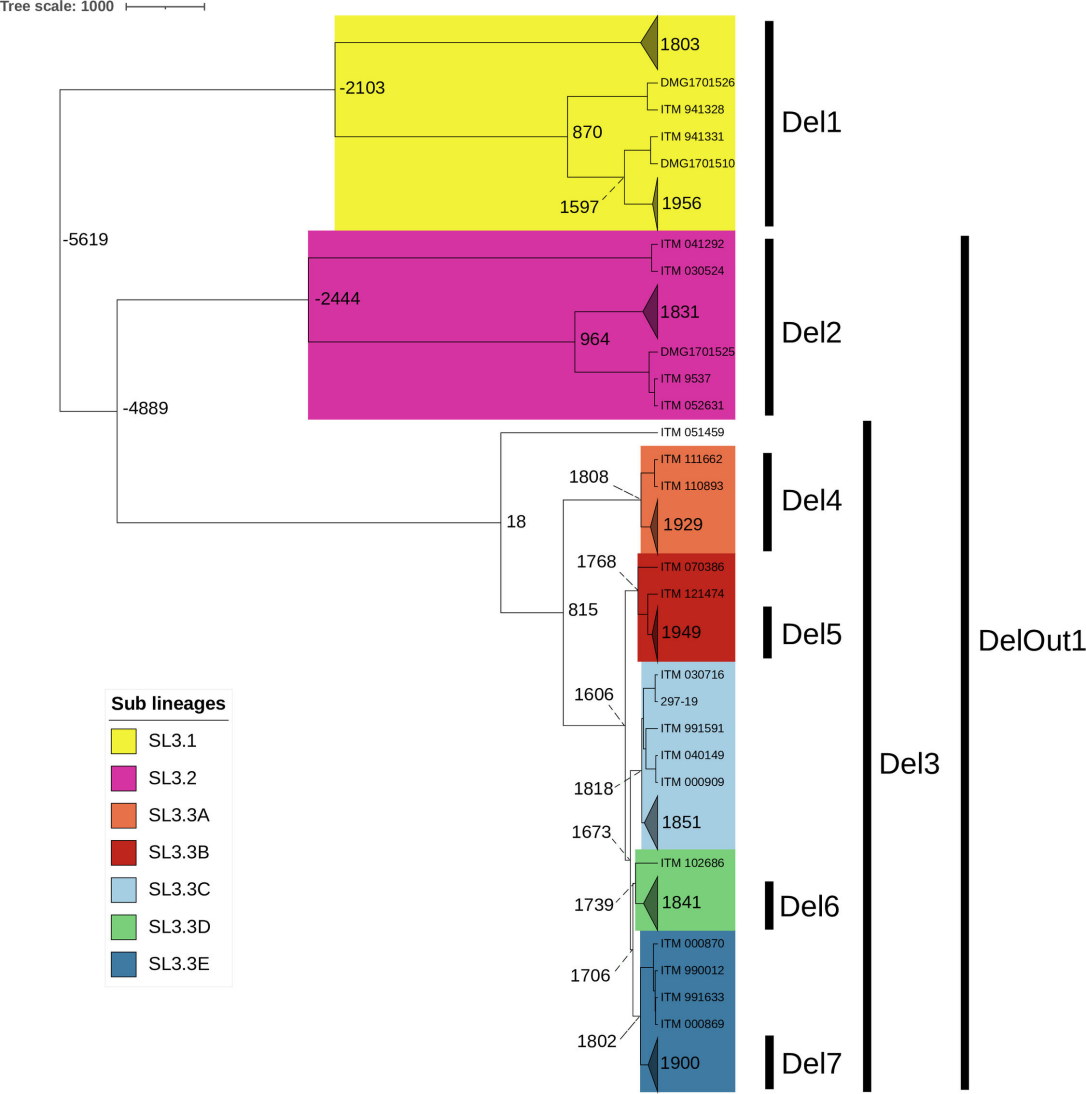
Bayesian temporal analyses on a representative 300 isolate data set of *Mu’*s recent lineage. BEAST-dated phylogenetic tree of a subset of 300 *Mu* strains built from non-recombining SNPs. Mean node ages are indicated in calendar years. The branches of the different groups are collapsed and colored. An outgroup was included but not represented. The youngest and oldest isolates of this data set date to 1945 and 2019, respectively.

There was a difference of 630,000 base pairs between the smallest and largest of the assembled genomes. We therefore investigated the relationship between genome size and group membership. Surprisingly, the genomes of SL3.3A appeared to be smaller than those of the other clades, whereas genome size was highly heterogeneous in SL3.1, SL3.2, and SL3.3E (Fig. 7). We then identified specific sequences common to the isolates of a given group (sub-lineage or clade) but not present in the isolates of the other ones (Fig. 5A; Table 4). To achieve this, we removed the atypical isolates from the first node of each of the five clades. First, we identified one specific sequence, absent from all Beninese and Nigerian strains in clade SL3.3E and present in all other *M. ulcerans* strains in data set 2, which we named Del7 (Table 4). A blast analysis of the sequences surrounding this deletion showed the region to be very well conserved, without rearrangements. We observed a specific loss of 481 bp corresponding to a sequence straddling two loci, MUL_0115 (MUL_RS00990) and MUL_0116 (MUL_RS00995), encoding the large (CoxL) and small (CoxS) chains of carbon monoxide dehydrogenase, respectively (Table 4). This deletion was common to all the strains from SL3.3E with Benin and Nigeria origin: the strains of the eight genotypes described here and the other SL3.3E strains from previous studies. Second, we identified similar specific sequences to the other clades named Del4, Del 5, and Del6 (Table 4), except the SL3.3C clade, for which no specific sequences were found. The SL3.3A strains, which had the smallest genomes, presented the largest deletion, of about 20,000 bp (Del4a and Del4b) (Table 4). The atypical strains at the junction of each clade from SL3.3A to SL3.3E did not share the specific deletion identified (Fig. 5 and 8). Third, at the level of the three sublineages SL3.1, 3.2, and 3.3, we similarly identified specific sequences, named Del1, Del2, and Del3 (Table 4). Finally, the sub-lineage SL3.1 is composed of Australian strains present also a specific sequence that is only present in this sub-lineage and named DelOut1 (Table 4). As this sequence is also present in the *M. marinum* genome, it probably corresponds to a common deletion occurring in the SL3.2 and SL3.3 sub-lineages. The impact of the traits encoded by these specific sequences on the differential ecology or virulence of the strains concerned remains unknown but all the sub-lineages comprised isolates pathogenic in humans.

### Emergence and worldwide spread of the recent *Mu* lineage over the last centuries (data set 2)

We also performed molecular dating analyses on data set 2 to gain insight into the evolution and spread of this species. We performed temporal analyses on a subset of 300 genomes, selected with Treemmer, representative of *Mu’*s recent lineage genetic, temporal, and geographic diversity (Fig. 8; Table 5). We used an average core genome substitution rate of 7 × 10^−8^ substitutions per site per year, consistent with previous findings (39) and the present study. We estimate that *Mu’*s recent lineage originated about 7,600 years ago (−5616; 95% HPD, −23,153/−1,037). From this period, different tree nodes occurred and led to the emergence of the three geographically distinct sub-lineages corresponding to the epidemiologic outbreak observed in populations. The MRCA was estimated for each *Mu* sub-lineage and clade, and the dates obtained are close to those reported in previous studies if already estimated (Table 5). Furthermore, the estimated MRCA date of SL3.3E obtained with the analysis performed on data set 2 (1900; 95% HPD 1707–1967) is close to the one obtained on data set 1 (1893; 95% HPD: 1826–1941). The sequences of events leading to the emergence of the sub-lineages and clades, long before European colonization, and the primary natural reservoir of *Mu* remain unknown.

**TABLE 5:** Estimation of the divergence dates from the most recent common ancestors of the *Mu’*s recent lineage (data set 2)

			MRCA (95%HPD)	MRCA from previous study (95% HPD)	Reference for previous estimated MRCA
	Worldwide	Lineage 3	−5619 (−23153/–1037)	NA	NA
Australia	Australia, Victoria	SL3.1	1803 (1365–1947)	1766 (1646–1877)	(39)
		SL3.2-SL3.3	−4889 (−13524/–962)	NA	NA
	PNG and Africa	SL3.2	964 (–623/1701)	865 (226–1324)	(40)
Mu-A2	Africa	SL3.2	1831 (1567–1943)	1800 (1689–1879)	(40)
Mu-A1	Africa	SL3.3A to SL3.3E	815 (−1262/1548)	702 (24–1199)	(40)
Mu-A1	Nyong-Cameroon	SL3.3A	1929 (1779–1987)	1901 (1848–1937)	(40)
		SL3.3B to SL3.3E	1606 (937–1831)	NA	NA
Mu-A1	Mape-Cameroon	SL3.3B	1949 (1842–1994)	2001 (1995–2006)	(41)
		SL3.3C to SL3.3E	1673 (1186–1854)	NA	NA
Mu-A1	Ghana/Cote d’Ivoire/Benin	SL3.3C	1851 (1613–1942)	NA	NA
		SL3.3D- SL3.3E	1706 (1270–1868)	NA	NA
Mu-A1	DRC	SL3.3D	1841 (1605–1936)	1865 (1803–1915)	(42)
Mu-A1	Benin/Nigeria	SL3.3E	1900 (1707–1967)	1890 (1835–1932)	(40)

^*a*^ NA- Not applicable.

## DISCUSSION

A better understanding of pathogen emergence, diversification, and evolutionary history is essential to improve the interpretation of population dynamics and pathogen diversity, and for effective disease surveillance (43). To this aim, this study brings together more genomes of the recent lineages than any other previous study, which independently investigated the phylogeny of *Mu* strains circulating in particular regions of Africa or Australia (39–42, 44, 45). One of the major conclusions of previous studies was a random distribution of the genotypes circulating at the local level. Importantly, we did not find this to be the case both in our previous study (24) and in this work, with a specific distribution of Mu-A1genotypes observed at the local scale for 307 isolates from a particular well-known endemic area. The genotypes specific to the Oueme River area were the latest to emerge. Based on our spatial clustering analysis, our data suggest that *M. ulcerans* was initially present in South-West Nigeria, where it spreads from east to west to reach the Oueme River. In fact, almost all patients reporting residence in Nigeria have a strain belonging to genotypes 1, 2, 3, or 6 (Table 1). We were even able to detect a difference in genotype distribution (genotypes 7 and 8) between the north and the south of the Oueme River, despite there being only 40 km between these two areas. On the contrary, the 12 Mu-A2 strains in this same endemic area did not present any specific spatial clustering.

We infer here that the Mu-A1 lineage (SL3.3) was introduced in Africa centuries ago (MRCA: 815; 95% HPD −1232/1548) and emerged in the form of endemic outbreaks in different African areas at different time points, from 1841 for SL3.3D in the Democratic Republic of Congo to 1949 for SL3.3B in the Mape region of Cameroon. These dates are in accordance with those previously estimated (Table 5) (40, 42). Sub-lineages, clades, and even genotypes are now firmly established at the local level, with localized persistence over decades, supported by the identification of specific sequences for each group. These results are consistent with the establishment of separate, locally entrenched sources of *Mu* with a limited flow of bacteria either within or between countries, in line with the country-specific nature of the groups described. We also confirm the strong association between particular clades and hydrological basins. For example, the isolates from the Oueme basin in Benin are phylogenetically close to those from South-West Nigeria, and both sets of isolates belong to the same clade SL3.3E. The isolates from the Kouffo basin in Benin, which is less than 100 km away from the Oueme basin, are related to a different clade (SL3.3C) corresponding to isolates from Ghana and Côte d’Ivoire, which are located much farther away (between 200 and 600 km).

We also identified specific genomic patterns associated with each sub-lineage or clade. These deletions may be caused by the expansion of insertion sequences (IS2404 and IS2606) in the genome of *Mu* leading to the inactivation of many genes, and ultimately, their deletions (4). To study this mechanism in more detail, long-read sequencing would be required. *Mu* is thought to evolve by clonal expansion without recombination. The proportion of recombining sites identified by ClonalFrameML is very low: 0.01% for data set 1 (561 bases / 4.5M) and 1.5% for data set 2 (71,726 bases /4.5M). The relative contribution of recombination over mutation on genetic diversity was measured by an R/Theta ratio of 0.08 (data set 1) and 0.05 (data set 2), as expected for a clonal expanding pathogen such *Mu*. Our observations are not suggestive of the bacteria originating in the Cameroon/Gabon area and then spreading outwards (7, 40). Indeed, a specific deletion in SL3.3A strains (Nyong-Cameroon and Gabon isolates) is the signature of this group, although only three strains from Gabon are available. In addition to the isolates of SL3.3C, which had no identified specific sequence, a small number of isolates at the first node of each clade were also atypical, with no specific sequence. Our suggestion based on the presence of these specific sequences is also confirmed by the pastML analysis performed on the data set 2 and presented in Fig. S2, where it is not possible to predict a common ancestral geographic location to the five clades of SL3.3. The origin of the spread of Mu-A1 (SL3.3) in Africa remains unclear and additional sequences will be required from *Mu* strains from all the African countries where this bacterium is endemic to address this specific question.

In the absence of transmission between humans, the non-random distribution of genotypes probably results from persistent reservoirs in the aquatic environment, in which *Mu* can reside for several years (46, 47). Moreover, there are several lines of epidemiologic evidence to support these observations, with changes in the hydrological features of the landscape linked to BU outbreaks. For example, the construction of a dam on the Mape River in Cameroon (1985–1988) coincided with the emergence of the specific SL3.3B isolates in this area (41). It remains unclear how *Mu* was introduced into new environments and then expanded independently. A role for terrestrial wild mammals or humans has been suggested but other hypotheses include introduction and transmission *via* aquatic insects, migrating animals (such as bats and birds), and the livestock trade. The presence of the SL3.2 sub-lineage strains (Mu-A2) at very low frequency (0.6%–2% of all isolates) throughout the African countries endemic for Buruli ulcer remains a mystery, these isolates belonging to the same sub-lineage as Papua New Guinea strains (MRCA: 964) (7). Finally, it has been suggested that the Australian isolates evolved from an ancestral progenitor from Papua New Guinea (39) but our results are not consistent with this hypothesis, supported also by the pastML analysis (Fig. S2).

Precise outbreak investigations at a local scale, including analyses of human behaviors, living spaces, and the environment, would probably facilitate the identification of sources of contamination and transmission routes, which remain unclear for *Mu*, as for other environmental bacterial pathogens (43, 48). We plan to perform such investigations, focusing on the genotypes circulating specifically in the southeastern part of the Oueme River basin. We have identified genotypes specific to a small geographic area (radius: 25 km) that had recently (1987) diverged. This is the ideal place in which to pursue an in-depth survey of the population to identify the likely sources of contamination. The identification of sources of contamination and reservoirs in Africa could facilitate significant long-term decreases in *Mu* transmission.

## Data Availability

The following link https://doi.org/10.57745/G5TBJA contains control files (full BEAST control files and other related data as ClonalFrameML files).

## SUPPLEMENTAL MATERIAL

Fig. S1 spectrum.03827-23-s0001.tif Structure and position of *M. ulcerans* lineage 3.

Fig. S2 spectrum.03827-23-s0002.tif Phylogeography of lineage 3.

Table S1 spectrum.03827-23-s0003.xlsx Description of the samples used in the study.

ASM does not own the copyrights to Supplemental Material that may be linked to, or accessed through, an article. The authors have granted ASM a non-exclusive, world-wide license to publish the Supplemental Material files. Please contact the corresponding author directly for reuse.
