# Supplementary figures and images for: Emergence and spread of Mycobacterium ulcerans at different geographic scales
Source: Microbiol Spectr. 2024 Mar 5;12(4):e03827-23. doi: 10.1128/spectrum.03827-23 (PMC10986537; doi:10.1128/spectrum.03827-23)

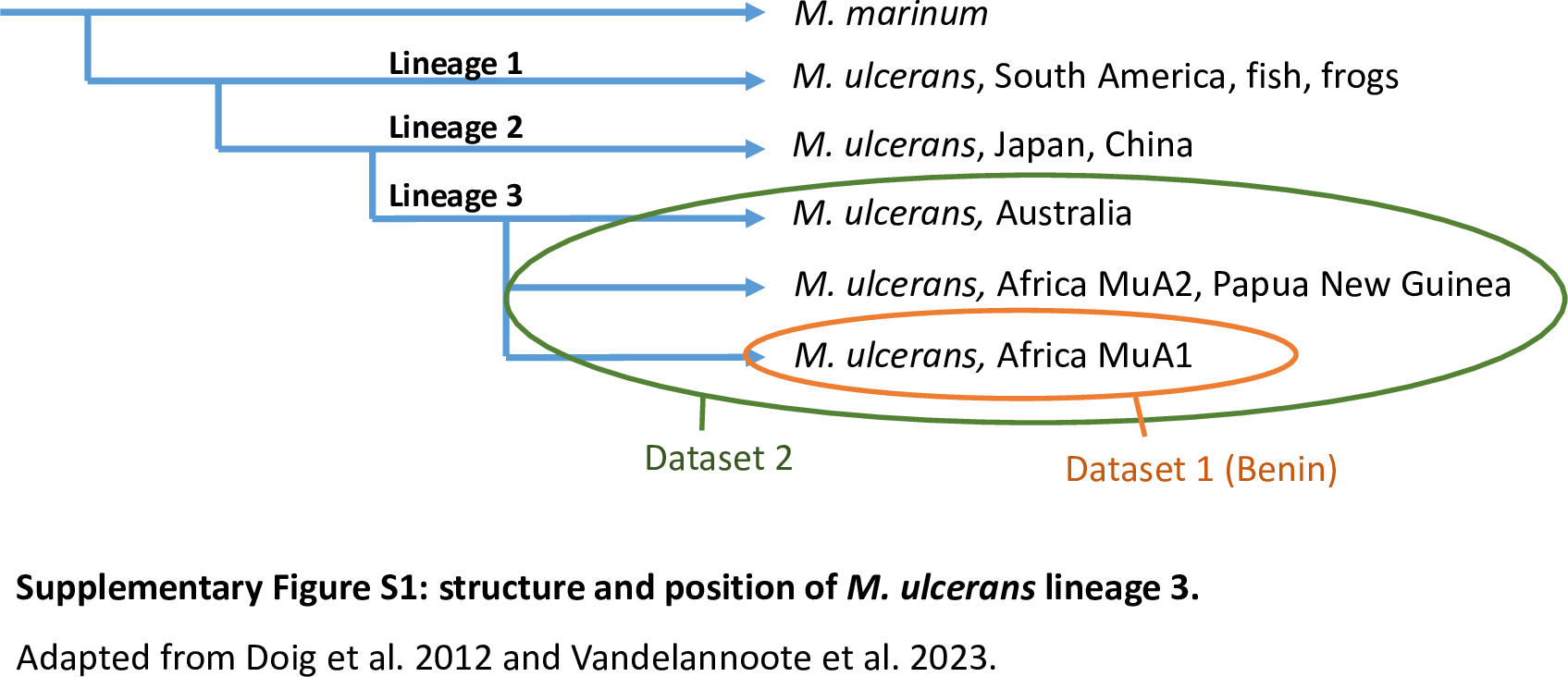

Supplement: Fig. S1 — Structure and position of M. ulcerans lineage 3. [file spectrum.03827-23-s0001.tif]

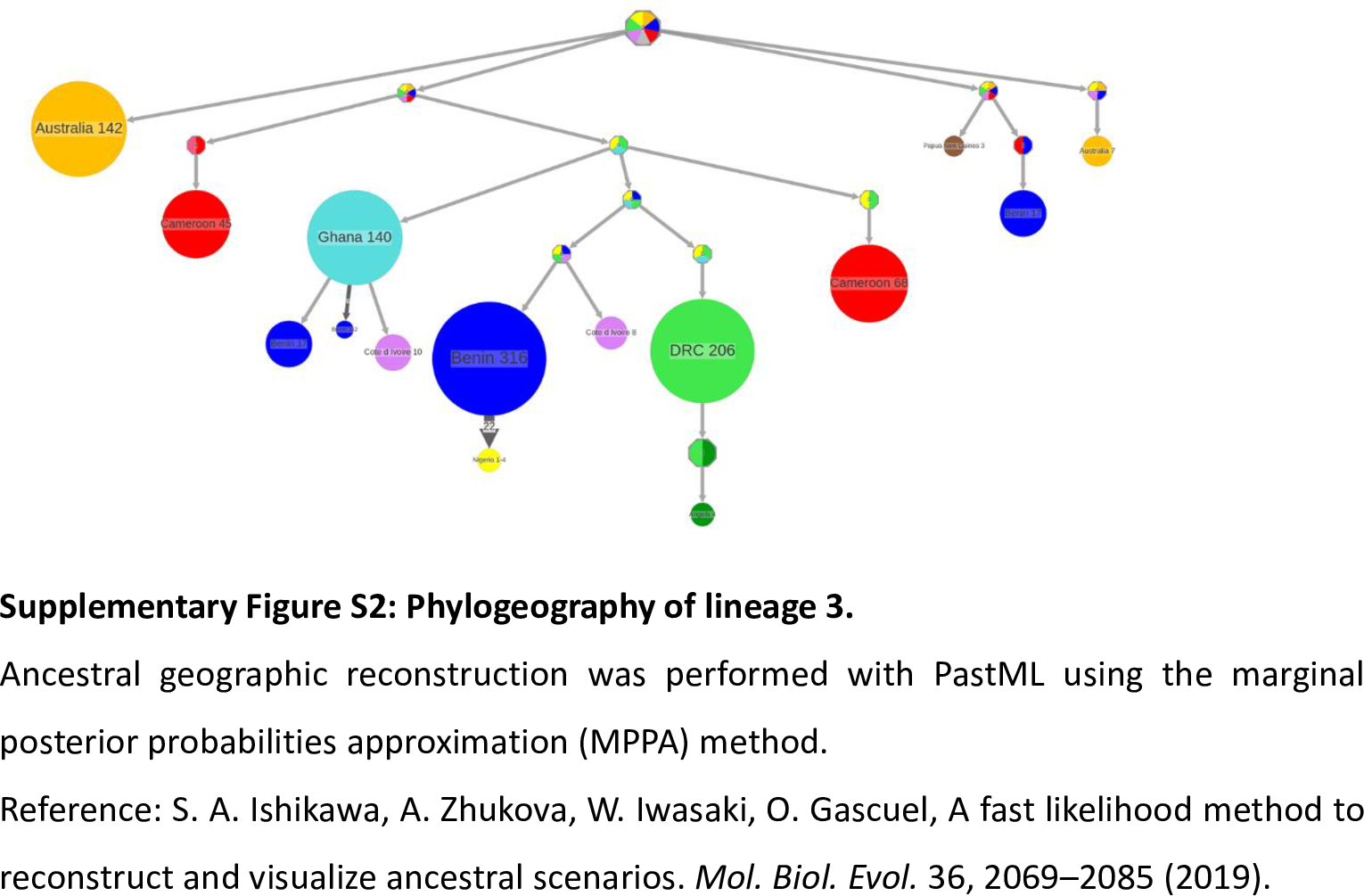

Supplement: Fig. S2 — Phylogeography of lineage 3. [file spectrum.03827-23-s0002.tif]
